# TPS46, a Rice Terpene Synthase Conferring Natural Resistance to Bird Cherry-Oat Aphid, *Rhopalosiphum padi* (Linnaeus)

**DOI:** 10.3389/fpls.2017.00110

**Published:** 2017-02-03

**Authors:** Yang Sun, Xinzheng Huang, Yuese Ning, Weixia Jing, Toby J. A. Bruce, Fangjun Qi, Qixia Xu, Kongming Wu, Yongjun Zhang, Yuyuan Guo

**Affiliations:** ^1^State Key Laboratory for Biology of Plant Diseases and Insect Pests, Institute of Plant Protection, Chinese Academy of Agricultural SciencesBeijing, China; ^2^Institute of Plant Protection, Jiangsu Academy of Agricultural SciencesNanjing, China; ^3^College of Plant Protection, Shandong Agricultural UniversityTai’an, China; ^4^Department of Biological Chemistry and Crop Protection, Rothamsted ResearchHarpenden, UK

**Keywords:** insect–plant interactions, *Rhopalosiphum padi*, *tps*46, (*E*)-β-farnesene, terpene syntheses

## Abstract

Plant terpene synthases (TPSs) are key enzymes responsible for terpene biosynthesis, and can play important roles in defense against herbivore attack. In rice, the protein sequence of TPS46 was most closely related to maize TPS10. However, unlike maize *tps10*, *tps46* was also constitutively expressed in rice even in the absence of herbivore attack. Potential roles or constitutive emissions of specific volatiles may due to the constitutive expressions of *tps*46 in rice. Therefore, in the present study, RNA interference (Ri) and overexpression (Oe) rice lines were generated to investigate the potential function of TPS46 in *Oryza sativa* sp. *japonica*. Interestingly, the rice plants become more susceptible to *Rhopalosiphum padi* when expression of *tps*46 was silenced compared with Wt in greenhouse conditions. Artificial infestation bioassays further confirmed that Ri rice lines were susceptible to *R. padi*, whereas Oe rice lines were repellent to *R. padi*. Based on GC-MS and ToF-MS analysis, a total of eight volatile products catalyzed by TPS46 in rice were identified. Among them, only limonene and Eβf could be detected in all the Ri, Oe, and Wt lines, whereas other six volatiles were only found in the blend of volatiles from Oe lines. Moreover, the amount of constitutive limonene and Eβf in the Ri lines was significantly lower than in Wt lines, while the amounts of these two volatiles in the Oe line were obviously higher than in control rice. Our data suggested that the constitutive emissions of Eβf and limonene regulated by the constitutive expression of *tps*46 may play a crucial role in rice defense against *R. padi.* Consequently, *tps*46 could be a potential target gene to be employed for improving the resistance of plants to aphids.

## Introduction

Plant terpenoids have ecological functions in mediating plant interactions and in allowing them to withstand biotic and abiotic stress (Pichersky and Gershenzon, 2002; Degenhardt et al., 2003). Volatile terpenes can help plants attract pollinators and predators of herbivores (Pichersky and Gershenzon, 2002; Degenhardt et al., 2003; Lou et al., 2005, 2006; Yuan et al., 2008). Additionally, terpenoid phytoalexins may be involved in the defense against herbivores (Balkema-Boomstra et al., 2003). When attacked by a herbivore, many plants are able to initiate indirect defenses by synthesizing and releasing complex blends of volatile terpenes that attract natural enemies of the herbivore (Turlings et al., 1991; Turlings and Ton, 2006; Yuan et al., 2008; Degenhardt, 2009). In grasses, especially in rice and maize, the biosynthesis of volatile terpenes regulated by terpene synthase (*tps*) genes has been investigated in details (Degenhardt, 2009), and many grass TPSs play crucial roles in the biosynthesis of plant herbivore-induced volatiles, such as TPS1, TPS10, and TPS23 in maize (Schnee et al., 2002, 2006; Köllner et al., 2008), as well as Os02g02930, Os08g07100 (TPS46), TPS3, and Os08g04500 in rice (Cheng et al., 2007; Yuan et al., 2008).

In our previous work, we found that a candidate gene, *SSBis*039 (Os08g0167800), was significantly upregulated in rice after *Chilo suppressalis* (Lepidoptera: Pyralidae) infestation for 24 h (Sun et al., 2011). Because there was 100% identity between *SSBis*039 and *tps*46, we confirmed that *SSBis*039 was the rice *tps*46. Rice *tps*46 is known to be an important gene in defense against *Spodoptera frugiperda* (Lepidoptera: Noctuidae) and *C. suppressalis* (Yuan et al., 2008; Sun et al., 2011). Phylogenetic analysis has shown that rice TPS46 is very closely related to maize TPS10 (Yuan et al., 2008). To investigate the roles of TPSs, a full-length cDNA of *tps* was usually cloned from herbivore-damaged plants, and then expressed in *Escherichia coli* or overexpressed in *Arabidopsis thaliana* (Beale et al., 2006; Schnee et al., 2006; Nagegowda et al., 2008; Yuan et al., 2008). By using farnesyl diphosphate (FPP) as the substrate, *E. coli*-expressed recombinant maize TPS10 catalyzed the formation of 11 sesquiterpenes, including α-copaene, (*E*)-β-caryophyllene, (*E*)-α-bergamotene, sesquisabinene, (*E*)-β-farnesene (Eβf), germacrene, zingiberene, *α*-muurolene, β-bisabolene; δ-cadinene, and sesquiphellandrene (Schnee et al., 2006). However, only six volatile compounds, such as (*E*)-α-bergamotene, sesquisabinene, Eβf, zingiberene, β-bisabolene, and sesquiphellandrene were significantly increased in *tps*10-overexpresing transgenic *A. thaliana* (Schnee et al., 2006).

*Escherichia coli*-expressed recombinant rice TPS46 converted FPP into a blend of 14 sesquiterpenes: 7-epi-sesquithujene, sesquithujene, (*Z*)-α-bergamotene, (*E*)-α-bergamotene, sesquisabinene A, Eβf, sesquisabinene, γ-curcumene, an unknown sesquiterpene, zingiberene, β-bisabolene, β-curcumene, β-sesquiphellandrene, and (*E*)-γ-bisabolene (Yuan et al., 2008). Among these volatiles, (*E*)-α-bergamotene, sesquisabinene, Eβf, zingiberene, β-bisabolene, and sesquiphellandrene were the same volatile compounds synthesized by both TPS10 and TPS46 *in vitro* (Schnee et al., 2006; Yuan et al., 2008). Interestingly, these six specific volatile compounds were also the main products in the *tps*10 overexpressed *A. thaliana* (Schnee et al., 2006). However, the products regulated by TPS46 in rice plants were still unclear. Therefore, given the differences in products in different expression systems, intensive study (overexpression of *tps*46 in rice) was required in the present study to determine whether the products of TP*S*46 *in vitro* are consistent with that *in vivo.*

Previous studies revealed that *tps*10 was not expressed in maize in the absence of herbivore attack (Schnee et al., 2006), whereas the expression of *tps*46 could be detected in rice even if the plants were not stimulated by any external factors (Yuan et al., 2008; Sun et al., 2011). Therefore, *tps*46 may play a different biological role in rice under the natural conditions. In recent years, RNA interference (RNAi) technique is widely used in transgenic plants to explore the function of target genes. In the present study, RNAi and overexpression trials were conducted to silence or overexpress *tps*46 in rice (*Oryza sativa* ssp. *japonica* “Nipponbare” NPB), and then investigate the biological roles of TPS46 under natural conditions or overexpression conditions. Consequently, our results provide an important foundation for understanding the function of TPS46 in rice sesquiterpenoid biosynthesis and plant defense.

## Materials and Methods

### Isolation of cDNA of Rice *tps*46

The full-length cDNA of rice *tps*46 [previously named as *SSBis*039 and shown to be induced by *C. suppressalis* larvae (Sun et al., 2011)] was cloned from “NPB” rice leaf tissues using the SMARTer^TM^ RACE cDNA Amplification Kit (Clontech, Palo Alto, CA, USA) according to the protocol described in Sun et al. (2014). Two 5′GSPs (5′GSP1 and 5′GSP2) and two 3′GSPs, (3′GSP1 and 3′GSP2) were designed using Primer 5.0 software based on the partial *tps*46 sequence obtained by our previous study (Sun et al., 2011), and primers were listed in **Table 1**.

**Table 1:** Primers used in the study.

Primer name	Sequence (5′–3′)
***tps*46 *fragment clone***	
RiTPS46-forward	TCAggtaccactagtCTACACGAAATGAAGCCATA
RiTPS46-reverse	CATggatccgagctcAGTTCCAGGTGTTCCTCTAT
***tps*46 *gene clone***	
OeTPS46-forward	CATGTCATCGACACCTGCA
OeTPS46-reverse	TTAAATGCTATATGGCTCAACG
***qRT-PCR***	
TPS46-forward	TGAAGAGGCACTAGGTCCAAAC
TPS46-reverse	CCATCCCAACTAAAGAAGCACA
EF1α-forward	AGACGCACATCAACATCG
EF1α-reverse	GAACTTCCACAGGGCAATA
***Race-PCR***	
TPS46-3′GSP1	CACACGATGGTGGAAAGAGCTTAACGTTG
TPS46-3′GSP2	GACAGGAGCATGCTCGGAGCCCCATTA
TPS46-5′GSP1	CAAGCATCATGCTCTCCTCGGTTGTAGC
TPS46-5′GSP2	TGGACCTAGTGCCTCTTCAAATGAATCAA

*The restriction sites used for clone are underlined and shown in bold*.

### The *tps*46-RNAi and *tps*46-Overexpression Transgenic Trials

Based on the sequence of *tps*46 (Genbank accession number: EU596452), the rice *tps*46 fragment was amplified using the RiTPS46F and RiTPS46R primers (**Table 1**). The 535 bps PCR product was digested with both *Sac*I and *Spe*I or with *Kpn*I and *BamH*I, gel purified, and then ligated into the same restriction sites within the RNAi ptck303 binary vector of rice (Wang et al., 2004). The ptck303-*tps*46 RNAi binary vector contained an antisense *tps*46 fragment, a rice intron, and sense *tps*46 fragment between the maize (*Zea mays*) ubiquitin promoter and the nos 3′-terminator in the ptck303 binary vector (Wang et al., 2004; Kong et al., 2006). For *tps*46 overexpression, the full-length CDS of *tps*46 was amplified using the OeTPS46F and OeTPS46R primers (**Table 1**). Then, the 1641bp of *tps*46 was ligated to a pCXUN vector with the TA cloning system (Chen et al., 2009). Rice callus was induced from the embryos of mature seeds of the Japonica cultivars “NPB” using an *Agrobacterium tumefaciens*-mediated method (Qu et al., 2006; Park et al., 2012). Finally, more than 15 T0 *tps*46-RNAi (Ri) rice lines and 15 T0 *tps*46-Overexpression (Oe) lines were obtained. The segregation lines of Ri and Oe transgenic plants were set as RWt or OWt controls.

### Insect Population Survey on T1 Ri Lines

Seven T1 Ri line (Ri1, 3, 4, 5, 8, 10, 11) and three T1 RWt line (RWt1, 2, 3) rice seedlings were first grown in a MS plus (75 mg L-1, Hygromycin B) selective liquid medium for 3 days in an incubator (28°C/26°C, 16 h of light/8 h of dark, and 50% humidity, respectively). Rice seedlings were grown individually in a cylindrical pot (25 cm and a height of 30 cm) in a greenhouse with temperature ranging from 24 to 32°C, 16 h of light/8 h of dark, and 50% humidity, where aphids, such as *Rhopalosiphum padi* (Hemiptera: Aphididae), *Myzus persicae* (Hemiptera: Aphididae), *Brevicoryne brassicae* (Hemiptera: Aphididae), and *Aphis gossypii* (Hemiptera: Aphididae), occurred naturally. The quantitative real-time PCR (qRT-PCR) measurements were conducted to investigate the target mRNA transcripts in leaves and sheaths of rice seedlings at the tillering stage. After qRT-PCR, every rice line as one treatment was random placed in a greenhouse to evaluate the insect infestation when exposed to aphids. All treatments in the experiment were repeated three times. This survey experiment was repeated twice. Insect infestation was recorded every day between 15:00 and 17:00 from tillering to the grain-filling stage of the rice plants. Aphid species were identified in insect taxonomy and toxicology laboratory, China Agricultural University.

### Bioassay of *R. padi* Performance on Plants

Newly emerged wingless *R. padi* colonies were kindly provided by Professor Xi-wu Gao of the insect toxicology laboratory, China Agricultural University, which had been maintained in the absence of insecticide exposure more than 10 years (Lu et al., 2013). The different rice lines were cultivated as described in the previous section “insect population survey on T1 Ri lines.” Before bioassay, expression of *tps*46 in the Ri3, Ri5, Ri8, Ri10, RWt, Oe6, Oe7, Oe9, Oe11, and OWt rice lines at jointing-booting stage was evaluated with qRT-PCR measurements. Soon after, Ri3, Ri5, Ri8, Ri10, Oe6, Oe7, Oe9, and Oe11 were selected for indoor bioassays in an insect-free greenhouse (**Figure 2** and **Supplemental Figure S1**). The temperature in the greenhouse was kept at 22–28°C and artificial infestation was performed between 13:00 and 17:00 PM. Each rice seedling in each pot was infested with 40 newly emerged wingless *R. padi*, and the number of *R. padi* on each rice seedling was recorded after 24, 48, and 72 h. Each treatment was replicated 3–5 times.

### qRT-PCR Analysis of *tps*46 in Different Rice Strains

Total RNA from the leaves and sheaths of rice seedlings (Oe, Ri, and Wt) was extracted by using the SV Total RNA Isolation System (Promega, Madison, WI, USA). One microgram of total RNA was used as a template for synthesizing the first-strand cDNA following the manufacturer’s protocol for MMLV Reverse Transcriptase (Promega, Madison, WI, USA). The cDNAs were treated with Ribonuclease H (TaKaRa, Tokyo, Japan) and quantified on a ND-1000 spectrophotometer (NanoDrop, Wilmington, DE, USA) at OD_260_ nm. The qRT-PCR was performed using the SYBR Premix Ex Taq Kit (TaKaRa, Tokyo, Japan) on a Bio-rad iCycler detecting system with SYBR green fluorescent dye. The rice EF-1α gene (Genbank accession number: AK061464) was used as an internal control to normalize the transcript levels of *tps*46 in each experiment (Jain et al., 2006), and the specific primers of the target gene and reference gene are listed in **Table 1**. The qRT-PCR reactions were replicated five times each treatment and non-template control (NTC) reactions were performed in triplicate. The 2^-ΔΔCt^ method was used to evaluate the relative expression of the target gene (Livak and Schmittgen, 2001).

### Volatile Collection

Rice lines, including Oe6, Oe7, OWt, Ri5, Ri8, and RWt at the beginning of jointing-booting stage were selected for volatile collection by an open head-space sampling system (Yu et al., 2010). Pots containing one rice plant were placed within a cylindrical type glass container (25 cm in diameter × 50 cm high). The container was sealed with a glass lid that had an air inlet and an air outlet. The container was tightly sealed with metal clamps on the lid. Air, purified by passage through an activated charcoal filter, was actively pumped through the container at a flow rate of 1 ml/min with a vacuum pump (Beijing Institute of Labor Instrument, Beijing, China). Volatiles were collected for 6 h on 60 mg of 60/80 mesh Tenax-TA (Shanghai ANPEL Scientific Instrument Company, Shanghai, China) in an 8-mm-diameter glass tube, which was directly connected to the outlet. All connections were made with Teflon tape. Three to five replicates were performed for each experiment (Huang et al., 2015). All collections were conducted from 11:00 AM to 17:00 PM. The collected samples were stored at 4°C for further analysis.

### Gas Chromatography-Mass Spectrometry (GC-MS) Analyses

Volatiles from Ri5, Ri8, and RWt rice lines were extracted with 300 μl of HPLC-grade hexane (Fisher Scientific, Fairlawn, NJ, USA), and 21 ng n-octane (Sigma-Aldrich, Oakville, Canada) was individually added as an internal standard. The collected samples were analyzed by Agilent 6890 GC coupled with an Agilent 5973 MS detector on a HP-5MS dimethylpolysiloxane column (30 m × 0.250 mm × 0.25 μm, Agilent Technologies, Palo Alto, CA, USA). One microliter of the sample was used for injection by using a 10 μl syringe (Hamilton, USA). The GC oven temperature was maintained at 40°C for 2 min and increased to 100°C at a rate of 6°C/min (hold for 1 min), increased again at a rate of 5°C/min to 150°C (hold for 1 min), increased to 250°C at a rate of 10°C/min, and finally maintained at 250°C for 5 min. Helium was used as the carrier gas at 1.0 ml/min. Tentative identifications were made by comparison of mass spectra (a) with mass spectra libraries (NIST and Department of Chemical Ecology, Göteborg University, Sweden) and (b) with the mass spectra and retention times of authentic samples obtained from Fluka, Sigma^1^.

Volatiles from the Oe6, Oe7, and OWt rice plants adsorbed on Tenax-TA were eluted with 300 μl HPLC-grade hexane (Fisher, Fairlawn, NJ, USA), and 2.586 ng of ethyl caprate (Sigma-Aldrich, Oakville, Canada) was individually added as an internal standard. Comprehensive two-dimensional gas chromatography/time-of-flight mass spectrometry (GC x GC-ToF, Pegasus 4D, LECO, USA) was performed to analyze the volatile samples. The GC inlet and transfer line were held constant at 250°C. A 1 μl injection was made onto column 1 (Rxi-5Sil MS, 30 m × 0.25 mm i.d. × 0.25 μm, Restek). The eﬄuent from column 1 was then transferred to column 2 (Rtx-200, 2 m × 0.180 mm i.d. × 0.2 μm, Restek). Column 1 was held at 50°C for 1 min followed by a two-step temperature increase, first to 150°C (at a rate of 5°C/min, hold for 2 min) and then to 250°C (at a rate of 10°C/min, hold for 2 min). Column 2 was held at 55°C for 1 min followed by a two-step temperature increase to 155°C (at a rate of 5°C/min with a 2 min hold) and then to 255°C (at a rate of 10°C/min, with a 2 min hold). The volatile products were identified by comparing their retention times and mass spectra areas to authentic standards (Gaquerel et al., 2009; Huang et al., 2015).

### Data Analysis

Differences of *tps*46 expression levels, *R. padi* numbers and volatile emission between different rice lines were analyzed using one-way ANOVA methods by SAS 9.0 software for Windows with Duncan’s new multiple range method (*P* < 0.05) (SAS 9.0 system for windows, 2002, SAS Institute Inc., Cary, NC, USA).

## Results

### *R. padi* Was the Dominant Aphid Species on the *tps*46-RNAi Rice Plants

**FIGURE 1:**
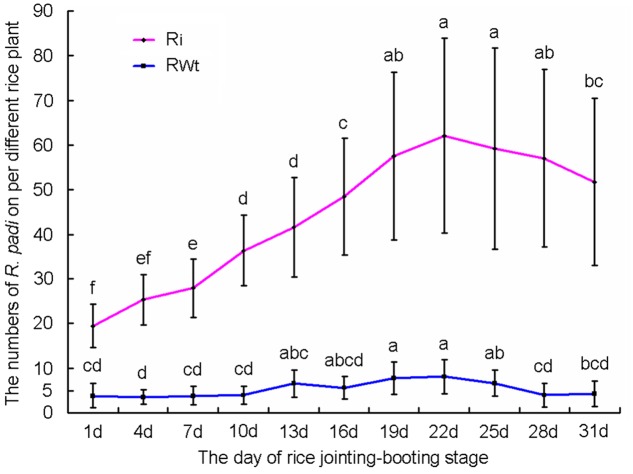
**The variation trends of *Rhopalosiphum padi* population numbers on T1 Ri line and RWt rice plants at 1–31 days of jointing-booting stage**. Different lowercase letters above each bar indicate statistical difference with a statistical analysis system (SAS) followed by the Duncan’s multiple comparison test (*P* < 0.05).

**FIGURE 2:**
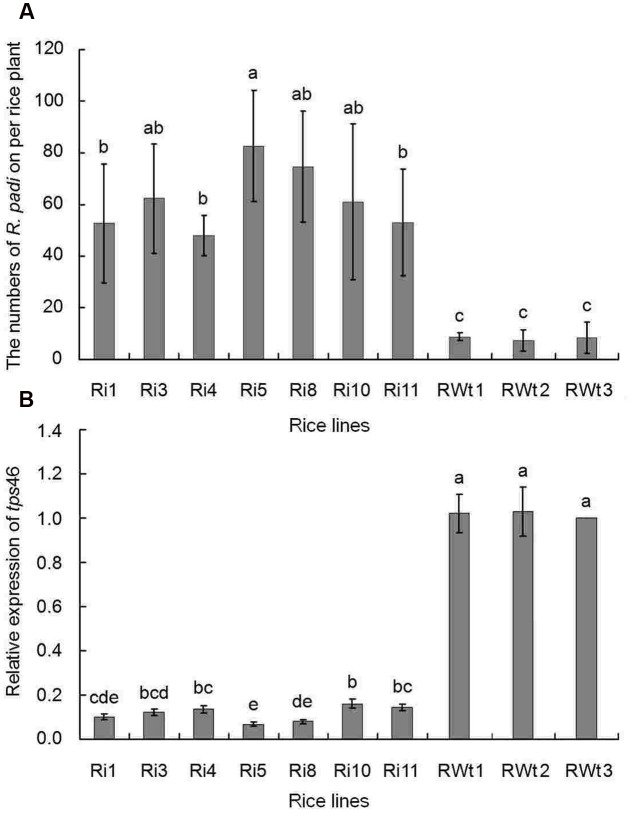
**The *R. padi* population numbers in different rice lines at the 22 days of jointing-booting stage**. Ri1, 3, 4, 5, 8, 10, and 11 were the positive T1 Ri rice lines; RWt controls were the segregation lines of Ri transgenic plants, and 1, 2, 3 were the seeds obtained from three different maternal rice. **(A)** The numbers of *R. padi* numbers on different rice species. **(B)** The relative expressions of *tps*46 in different rice species. Different lowercase letters above each bar indicate statistical difference with a SAS followed by the Duncan’s multiple comparison test (*P* < 0.05).

In our greenhouse, several aphid species on rice lines were observed and recorded, while only the significant difference in *R. padi* numbers between the Ri and RWt rice plants were observed. Other aphids tested included *M. persicae*, *B. brassicae*, and *A. gossypii*. In infestation trials, a small number of winged *R*. *padi* were found on rice plants in the late tillering stage. Then the wingless *R*. *padi* population sharply increased on Ri rice lines in the jointing-booting stage and the population reached a peak value (62 ± 22 aphid/ individual Ri plant) at 22 days into the jointing-booting stage (**Figure 1**). However, the *R*. *padi* population on the segregation lines of Ri transgenic plants (RWt) increased very little during the same stage and after 31 days was not significantly different the population size at the start (**Figure 1**). Also, the same tendency in this experiment was reproduced in another repeats (**Figure 1** and **Supplemental Figure S3**). Moreover, *R*. *padi* populations on all seven tested Ri rice lines were significantly higher than on control rice lines (*P* < 0.05). In contrast, there was no remarkable difference in *R. padi* populations among the three RWt control rice lines (*P* > 0.05) (**Figure 2A**). Expression levels of *tps*46 were significantly lower in all the seven tested Ri rice lines than in control lines (*P* < 0.05), and there was no visible difference between the RWt control rice lines (*P* > 0.05) (**Figure 2B**). The *R*. *padi* population (83 ± 22 aphid/ individual Ri plant) on Ri5 rice plants among these Ri rice lines was significantly higher than on Ri1, Ri4 and Ri11 rice lines (*P* < 0.05) (**Figure 2A**). Interestingly, the expression of *tps*46 in the Ri5 line was only 6.81 ± 0.79% of the expression in control rice lines (**Figure 2B**). In addition, our observations have shown that there was no *R. padi* on control rice lines in the field or the greenhouse for many years until the Ri rice plants were cultivated.

### Artificial Infestation Bio-Assays of Different Rice Lines

**FIGURE 3:**
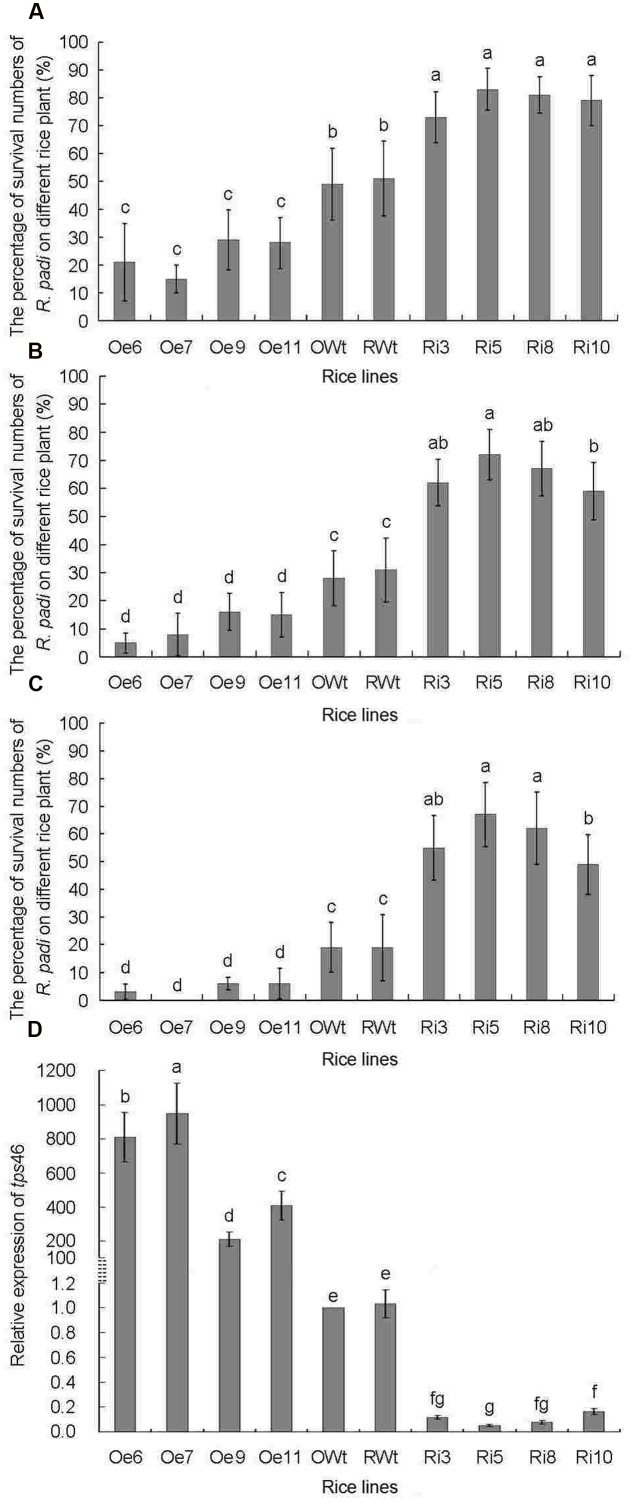
**The numbers of *R. padi* for indoor *R. padi* bioassay on different rice lines from 1 to 3 days of jointing-booting stage**. Ri3, 5, 8 and 10 were the positive T1 *tps*46-RNAi rice lines; Oe6, 7, 9 and 11 were the T1 positive *tps*46-Overexpression rice lines; RWt and OWt were the segregation lines of Ri and Oe transgenic plants. **(A)** The numbers of *R. padi* on different rice lines after 1 day treatments. **(B)** The numbers of *R. padi* on different rice lines after 2 days treatments. **(C)** The numbers of *R. padi* on different rice lines after 3 days treatments. **(D)** The relative expressions of *tps*46 in different rice lines before indoor *R. padi* bioassay. Different lowercase letters above each bar indicate statistical difference with a SAS followed by the Duncan’s multiple comparison test (*P* < 0.05).

The *tps*46 expression levels in different rice lines were analyzed by qRT-PCR before the *R*. *padi* infestation bio-assays. The expression of *tps*46 in all of the Oe rice lines was significantly higher than in the Ri and control rice lines (*P* < 0.05), whereas expression of *tps*46 in all of the Ri rice lines was obviously lower than expression in the control rice lines (*P* < 0.05). Additionally, there was no significant difference between the RWt and OWt control rice lines (*P* > 0.05) (**Figure 3D**).

The survival rate of *R. padi* on Ri rice lines was significantly higher than that on Oe and control lines (*P* < 0.05). The survival rate of *R. padi* on the Oe lines was significantly lower than the survival rate on control rice plants (*P* < 0.05), and there was no obvious difference in the *R. padi* survival rate between RWt and OWt control rice lines (*P* > 0.05) (**Figures 3A–C**). The survival rate of *R. padi* on the two control lines was only approximately 20% after 72 h. The survival rate of *R. padi* on the Ri lines was higher than 50% during the same period and reached 67 ± 11.5% on Ri5 rice plants (**Figure 3C**). During the same period, the survival rate of *R. padi* on the Oe rice lines was below 10%t after 72 h, and was less than 5% on the Oe6 and Oe7 rice plants (**Figure 3C**). Interestingly, the expression of *tps*46 in the Oe6 and Oe7 rice plants was 837 ± 144 and 976 ± 178 times higher than in control lines. The expression was significantly higher compared to expression in the other tested rice plants (*P* < 0.05). However, the expression of *tps*46 in the Ri5 lines was only 5.1 ± 0.94% of that in control rice lines (**Figure 3D**).

### Volatile Emission from Ri and Oe Rice Plants

**FIGURE 4:**
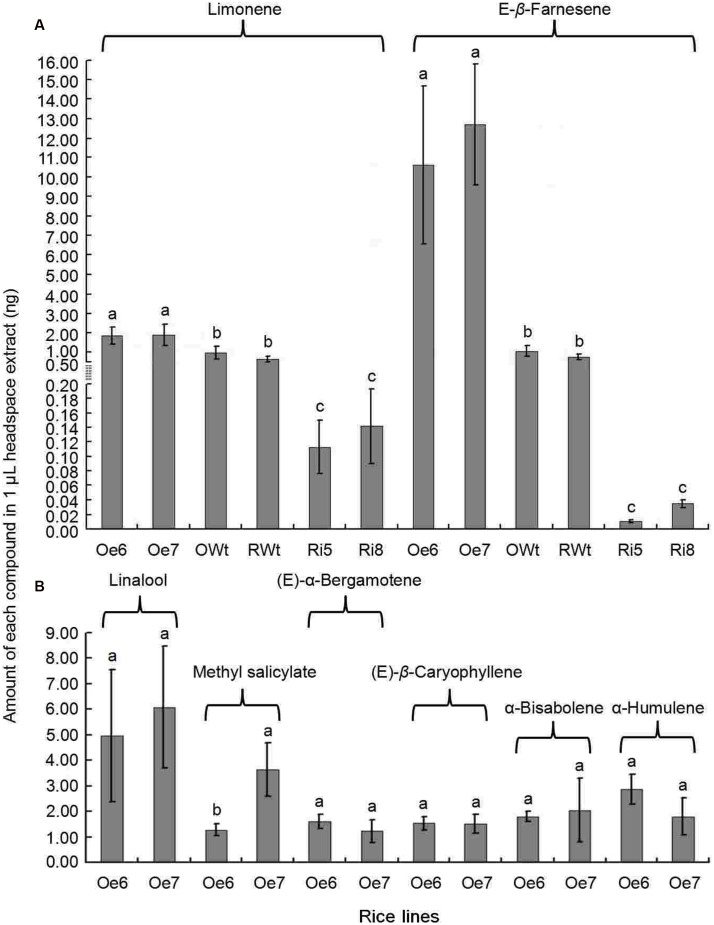
**A mount of each compound in 1 μL headspace extract of different rice lines**. Ri5 and 8 were the positive T1 Ri rice lines; Oe6, and 7 were the positive T1 Oe rice lines; RWt and OWt were the segregation lines of Ri and Oe transgenic plants. **(A)** Amount of limonene and (*E*)-β-farnesene in different rice lines. **(B)** Amount of linalool, methyl salicylate, (*E*)-α-bergamotene, (*E*)-β-caryophyllene, α-bisabolene, and α-humulene in different positive Oe transgenic rice lines. Different lowercase letters above each bar indicate statistical difference with a SAS followed by the Duncan’s multiple comparison test (*P* < 0.05).

**FIGURE 5:**
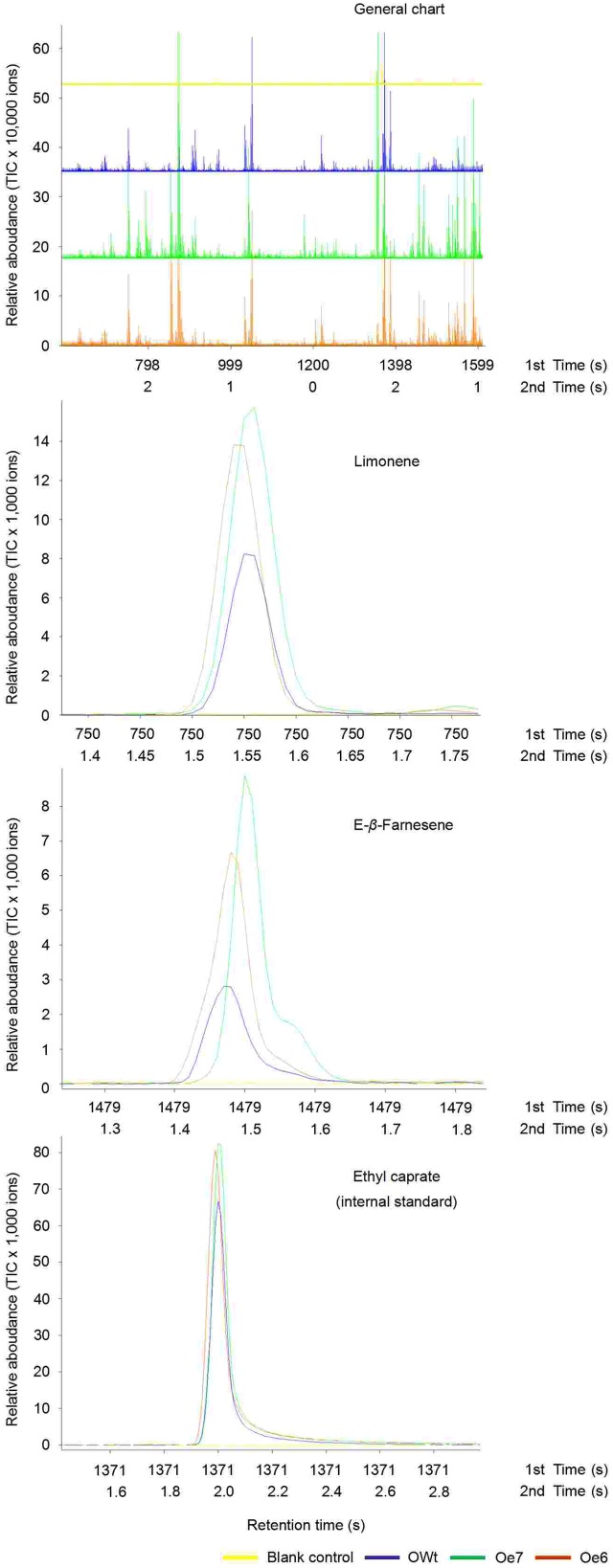
**Representative ToFMS of head-space volatile compounds from different rice lines at beginning of jointing-booting stage**. Oe6 and 7 were the positive T1 *tps*46-Overexpression rice lines; OWt were the segregation lines of Oe transgenic plants.

Recombinant TPS46 protein can catalyze substrates to produce several terpene volatiles *in vitro* (Yuan et al., 2008). In this study, we further investigated the volatile compounds released from the Ri, Oe, and Wt rice lines. GC-MS analyses indicated that the amount of two volatile compounds, limonene and Eβf, decreased significantly in the Ri rice lines compared to the RWt (control) rice plants (*P* < 0.05) (**Supplemental Figure S2** and **Figure 4A**). The amounts of limonene in 1 μL of extracts from Ri5, Ri8, and RWt rice headspace samples were 0.11 ± 0.04, 0.14 ± 0.05, and 0.65 ± 0.14 ng, respectively. The amounts of Eβf in Ri5, Ri8, and RWt rice headspace samples were 0.01 ± 0.002, 0.035 ± 0.005, and 0.77 ± 0.13 ng, separately (**Figure 4A**). As expected, the amount of limonene and Eβf increased in the Oe rice lines compared to the OWt plants (*P* < 0.05) (**Figures 4A** and **5**). The amounts of limonene in 1 μL extracts from the Oe6, Oe7, and OWt plants was 1.87 ± 0.42, 1.90 ± 0.53, and 0.99 ± 0.31 ng, respectively. Meanwhile, the amount of Eβf in the Oe6, Oe7, and OWt plants was 10.62 ± 4.04, 12.70 ± 3.09, and 1.06 ± 0.26 ng, respectively (**Figure 4A**). There was no significant difference in the amounts of limonene and Eβf between the OWt and RWt rice lines (**Figure 4A**).

**FIGURE 6:**
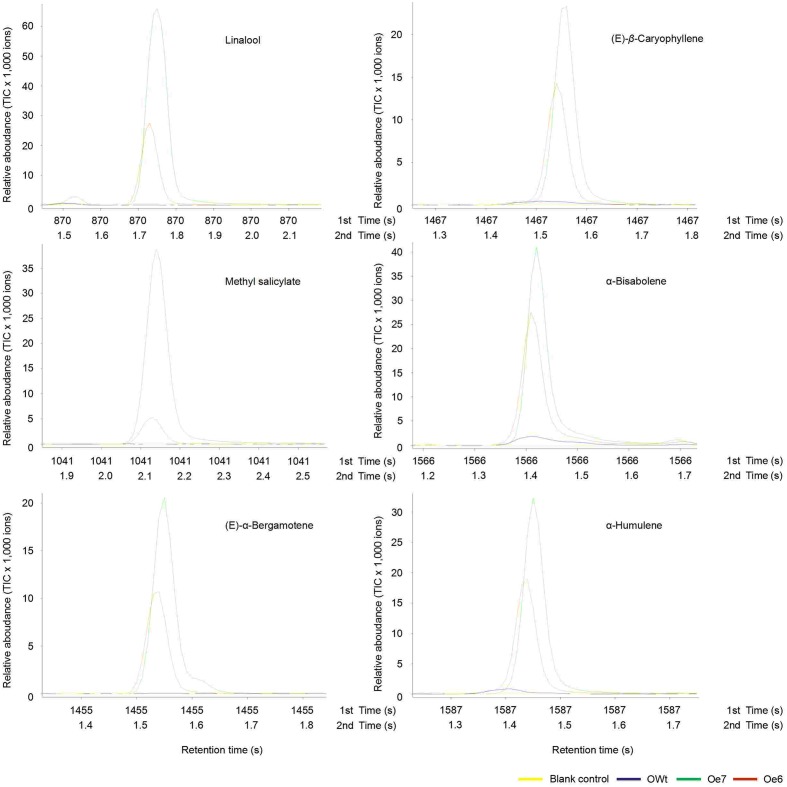
**Representative ToFMS of head-space volatile compounds from different rice lines at beginning jointing-booting stage**. Oe6 and 7 were the positive T1 *tps*46-Overexpression rice lines; OWt were the segregation lines of Oe transgenic plants.

Six other volatile compounds linalool, (*E*)-β-caryophyllene, methyl salicylate, α-bisabolene, (*E*)-α-bergamotene, and α-humulene were also increased significantly in the Oe rice lines compared to the OWt lines (*P* < 0.05). The content of these six volatiles in the OWt rice lines was extremely low (**Figure 6**). Also, these six volatile compounds were not detected in the Ri and RWt rice plants (**Supplemental Figure S2**). Among the six volatile compounds, the amount of linalool in 1 μL of extract from the Oe6 and Oe7 rice lines was 4.97 ± 2.58 ng, and 6.10 ± 2.38 ng, respectively (**Figure 4B**). The amount of (*E*)-β-caryophyllene, α-bisabolene, (*E*)-α-bergamotene, α-humulene in the Oe6 rice lines was 1.54 ± 0.27, 1.80 ± 0.19, 1.61 ± 0.29, and 2.88 ± 0.58 ng, separately. The amount of (*E*)-β-caryophyllene, α-bisabolene, (*E*)-α-bergamotene, α-humulene in the Oe7 lines was 1.53 ± 0.37, 2.05 ± 1.24, 1.24 ± 0.44, and 1.82 ± 0.71 ng, respectively (**Figure 4B**). Moreover, among the six volatile compounds, there were significant differences in the amount of methyl salicylate between the Oe6 and Oe7 lines (*P* < 0.05), and the amount of methyl salicylate in the Oe6 and Oe7 rice lines was 1.27 ± 0.24, and 3.65 ± 1.04 ng, respectively (**Figure 4B**).

## Discussion

The current study shows that rice *tps*46 (Os08g0167800) is a key gene responsible for biosynthesis of limonene, methyl salicylate, Eβf, (*E*)-β-caryophyllene, α-bisabolene, (*E*)-α-bergamotene, and α-humulene from FPP. Among these volatiles, constitutive emissions of limonene and Eβf may due to the constitutive expressions of *tps*46 under natural conditions in rice. Furthermore, it is shown that silencing expression of *tps*46 makes rice vulnerable to attack by a herbivore, *R. padi*, which does not usually attack wild type rice. This finding suggests that TPS46 plays an important role in rice innate immunity to aphids.

Three TPS enzymes (Os02g02930, TPS46, and Os08g04500) are involved in the synthesis of the main terpene released from *S. frugiperda-*damaged rice plants (Yuan et al., 2008). All of the volatile components catalyzed by the above three recombinant enzymes in the *E. coli*-expression system only appeared in the blend of rice volatiles induced by *S. frugiperda*. The volatile product of recombinant Os02g02930 was (*S*)-linalool, and the volatile components of recombinant Os08g04500 were β-elemene, (*E*)-β-caryophyllene, α-humulene, and germacrene (Yuan et al., 2008). However, the expression of *tps*46 was also found in untreated rice plants (Yuan et al., 2008; Sun et al., 2011). In our Ri and Oe trials, eight volatile compounds were confirmed to be regulated by TPS46 in rice plants (**Figures 5** and **6**; **Supplemental Figure S2**), and these compounds are known to be involved in the indirect defense of Gramineous plants against phytophagous pests (Degenhardt et al., 2003; Lou et al., 2005, 2006; Rasmann et al., 2005; Schnee et al., 2006; Yuan et al., 2008). Among these eight volatiles, six compounds, linalool, (*E*)-β-caryophyllene, methyl salicylate, α-bisabolene, (*E*)-α-bergamotene, and α-humulene were only emitted in *tps*46-overexpression lines. These six volatiles were also only found in the blend of volatile compounds from rice plants infested by *S. frugiperda* (Yuan et al., 2008). Interestingly, (*E*)-β-caryophyllene and α-humulene were the products of recombinant Os08g04500, whereas linalool was the main product of recombinant Os02g02930, and only (*E*)-α-bergamotene was the products of recombinant TPS46 (Yuan et al., 2008). In addition, suppression of emission of limonene and Eβf in Ri rice lines suggested that the biosynthesis of these two volatile compounds is regulated by *tps*46 in rice plants. Yuan et al. (2008) found that Eβf was the main product of recombinant TPS46 (Yuan et al., 2008). The results of this study revealed that the products of the same TPS in plants may be different from the TPS in the *E. coli*-expressed system. These differences could possibly be attributed to the complexity of terpene biosynthesis in rice. The constitutive emission of limonene and Eβf in untreated rice plants may be attributed to the expression of *tps*46 in the same plants.

It has been suggested that constitutive release of defensive volatiles should occur when plants are growing in an environment where there is a high probability of attack by herbivores (Degenhardt et al., 2003; Turlings and Ton, 2006). The constitutive release of volatiles is not only costly to plants, thereby resulting in yield declines but also inconsistent with the objectives of indirect defenses in plants because natural enemies do not get an “honest” signal indicating presence of prey (Degenhardt et al., 2003; Yamauchi et al., 2015). Nevertheless, there are several reports suggested that the release of volatiles is not necessarily very costly (Aharoni et al., 2005), and various plants constitute release some of the compounds of interests (Turlings and Ton, 2006). The nicest demonstration that constitutive emission might benefit pest control comes from the famous so-called “push-pull” studies by Khan et al. (1997), which also indicted that the constitutive emission volatiles from plants were also very important for plant defense against herbivore damage.

Although RWt and OWt were continuously cultivated in field or greenhouse for many years, few *R. padi* were found on these rice plants. In the present study, artificial infestation bioassays were consistent with the results of initial *R. padi* population survey in the greenhouse. After artificial infestation for 3 days, only approximately 20% of *R. padi* were observed on RWt and OWt rice lines, which indicated that they have some natural resistance to *R. padi*. Compared to control rice lines, *R. padi* significantly preferred to infest *tps*46-Ri rice lines (**Figures 1–3**). Differences in aphid infestation appear to be linked to differences in volatile production. For example, emission of two major volatiles, limonene and Eβf, was significantly reduced in the Ri lines (**Figure 4A**). These results indicate that limonene and Eβf may play a role in rice defenses against *R. padi*. However, to the best of our knowledge, there are almost no reports on roles of limonene in plant defenses against aphids. Our preliminary behavior assays also indicated that there was no obvious taxis response of *R. padi* to limonene stimuli at different doses (unpublished). Unlike limonene, Eβf was the main component of the alarm pheromone of *R. padi* (Nault and Bowers, 1974). In this work, the *R. padi* were significantly repelled by *tps*46-Oe rice lines (*P* < 0.05) (**Figures 2** and **3**), and the amount of Eβf was also significantly higher than the amount in control rice plants (*P* < 0.05) (**Figure 4A**). One hypothesis could be that Eβf has activity against aphids because it is the main component of the alarm pheromone for many aphids (Pickett et al., 2013). Eβf is secreted from the cornicle of aphids and causes other aphids in the vicinity to stop feeding, move away, and drop off the plant (Bernasconi et al., 1998; Hardie et al., 1999; de Vos and Jander, 2010). Genetic engineering technique could regulate plants to emit Eβf to repel aphids (Beale et al., 2006; Gao et al., 2015). However, in the current study, we found that Eβf was released with other compounds that might inhibit alarm pheromone activity (Bruce et al., 2005a) and constitutive emission of Eβf from transgenic wheat did not reduce aphid populations in field experiments (Bruce et al., 2015). Another possibility is that the blend of compounds released from wild type rice expressing *tps46* interferes with host recognition by the aphid (Bruce et al., 2005b).

In summary, the constitutive emission of Eβf and limonene from rice was regulated byTPS46. The *tps*46 was an important gene for rice defense against *R. padi.* Along with the development of insect-resistant transgenic plants (Wu et al., 2008), heterologous expression of TPS46 could be used as a potential strategy for improving the resistance of other crop species to aphids.

## Author Contributions

YZ, KW, and YG conceived and designed the experiments. YS, YN, and FQ performed transgenic rice experiments. YS, QX, and YZ performed bioassay of *R. padi* performance experiments. YS, XH, WJ, and YZ performed volatile collection and identification experiments; YS and YZ analyzed the data and wrote the paper; TB revised the paper.

## Conflict of Interest Statement

The authors declare that the research was conducted in the absence of any commercial or financial relationships that could be construed as a potential conflict of interest.

## Footnotes

1 http://www.sigmaaldrich.com/

## Supplementary Material

FIGURE S1 **The relative expressions of *tps*46 in different rice lines at the late tilling stage**. Oe1, 4, 6, 7, 8, 9 and 11 were the positive *tps*46-Overexpression rice lines; OWt were the segregation lines of Oe transgenic plants, and 1, 2, 3 were the seeds obtained from three different maternal rice plants. Different lowercase letters above each bar indicate statistical difference with a statistical analysis system (SAS) followed by the Duncan’s multiple comparison test (*p* < 0.05).

FIGURE S2 **Representative GC-MS of head-space volatile compounds from different rice lines at beginning of jointing-booting stage. (A)** Positive T1 *tps*46-RNAi line rice plants. **(B)** The segregation lines of Ri transgenic plants. **(C)** The standard sample of limonene. **(D)** The standard sample of (*E*)-β-farnesene.

FIGURE S3 **The variation trends of *Rhopalosiphum padi* population numbers on T1 Ri line and RWt rice plants at 1–31 days of jointing-booting stage with another repeats**. Different lowercase letters above each bar indicate statistical difference with a statistical analysis system (SAS) followed by the Duncan’s multiple comparison test (*P* < 0.05).
