# Supplementary figures and images for: TPS46, a Rice Terpene Synthase Conferring Natural Resistance to Bird Cherry-Oat Aphid, Rhopalosiphum padi (Linnaeus)
Source: Front Plant Sci. 2017 Feb 3;8:110. doi: 10.3389/fpls.2017.00110 (PMC5289981; doi:10.3389/fpls.2017.00110)

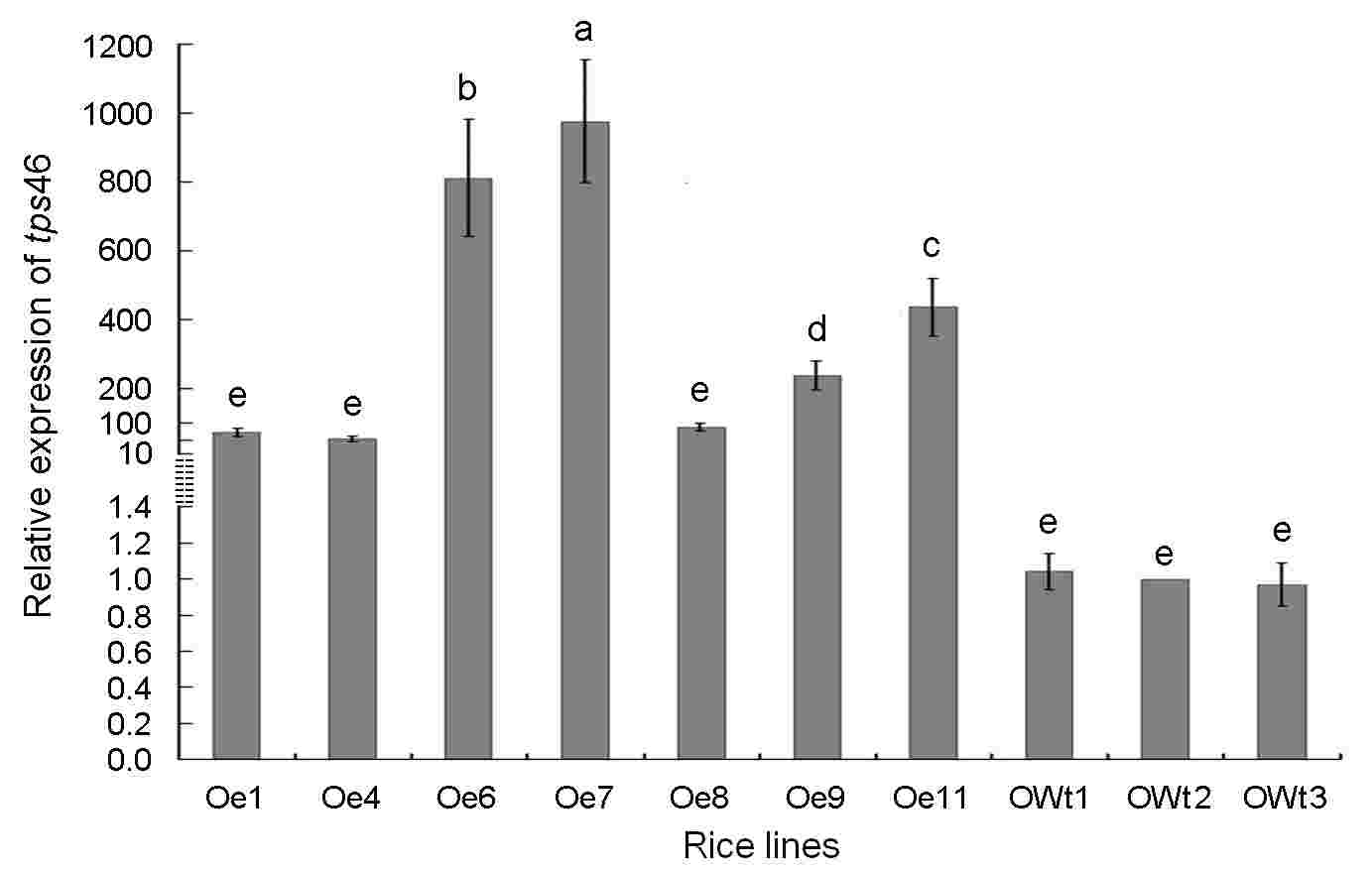

Supplement: FIGURE S1 — The relative expressions of tps46 in different rice lines at the late tilling stage. Oe1, 4, 6, 7, 8, 9 and 11 were the positive tps46-Overexpression rice lines; OWt were the segregation lines of Oe transgenic plants, and 1, 2, 3 were the seeds obtained from three different maternal rice plants. Different lowercase letters above each bar indicate statistical difference with a statistical analysis system (SAS) followed by the Duncan’s multiple comparison test (p < 0.05). [file Image_1.JPEG]

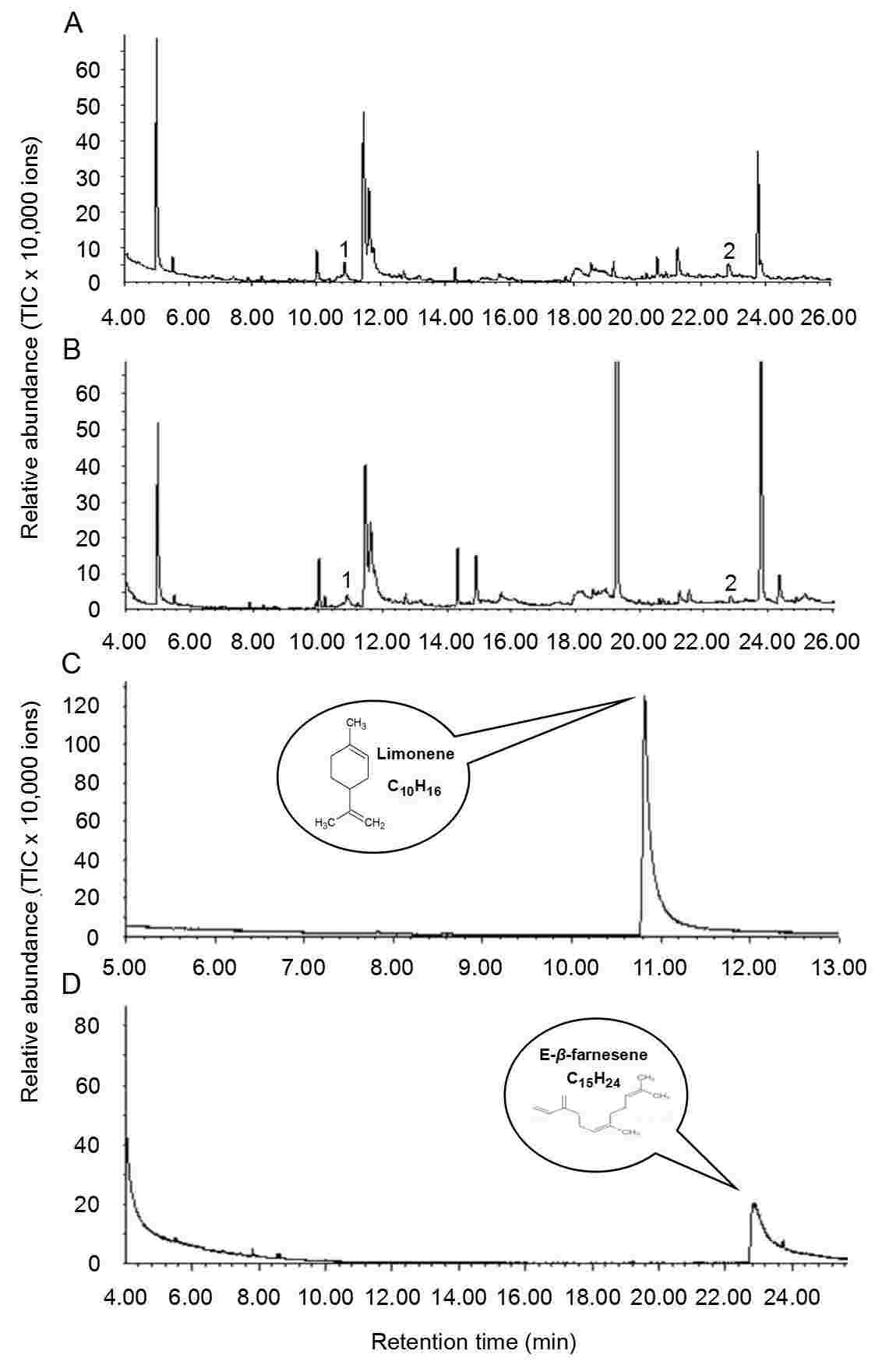

Supplement: FIGURE S2 — Representative GC-MS of head-space volatile compounds from different rice lines at beginning of jointing-booting stage. (A) Positive T1 tps46-RNAi line rice plants. (B) The segregation lines of Ri transgenic plants. (C) The standard sample of limonene. (D) The standard sample of (E)-β-farnesene. [file Image_2.JPEG]

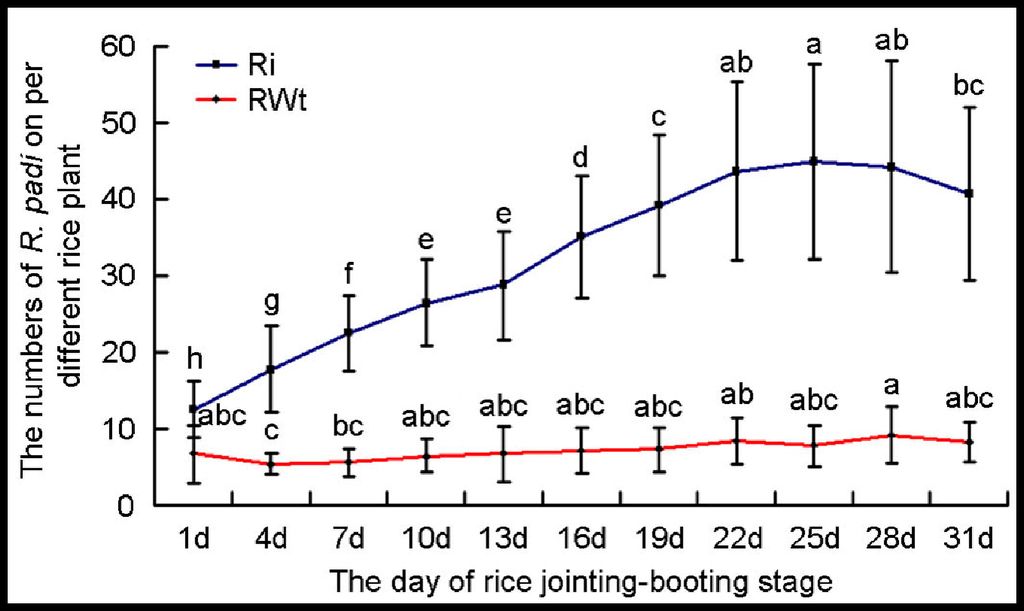

Supplement: FIGURE S3 — The variation trends of Rhopalosiphum padi population numbers on T1 Ri line and RWt rice plants at 1–31 days of jointing-booting stage with another repeats. Different lowercase letters above each bar indicate statistical difference with a statistical analysis system (SAS) followed by the Duncan’s multiple comparison test (P < 0.05). [file Image_3.JPEG]
